# Innovative Emerging Technologies for Pulse Flour Functionalization: A Comparison With Conventional Thermal and Biological Techniques

**DOI:** 10.1111/1541-4337.70581

**Published:** 2026-08-21

**Authors:** Federico Drudi, Silvia Tappi, Urszula Tylewicz

**Affiliations:** ^1^ Department of Agricultural and Food Sciences (DISTAL) University of Bologna Cesena Italy; ^2^ Interdepartmental Centre for Agri‐Food Industrial Research (CIRI AGRO) University of Bologna Cesena Italy

**Keywords:** fiber, legumes, nonthermal technologies, protein, soy, starch

## Abstract

Pulse flours, such as those from peas, beans, and chickpeas, are highly valued for their nutritional density, making them essential for meeting the rising global demand for plant‐based and gluten‐free foods. Despite these nutritional advantages, industrial use is limited by specific sensorial, nutritional, and technological constraints. Overcoming these challenges requires targeted functionalization strategies designed to impart improved and predictable techno‐functional properties. This review describes the characteristics of pulse flours and assesses both conventional (thermal, biological, and chemical) and innovative modification techniques, with particular emphasis on the application of nonthermal technologies and the main techno‐functional aspects. Thermal methods are the most studied, primarily inducing starch gelatinization and protein denaturation, generally improving water‐ and oil‐holding capacities but often reducing foaming and emulsifying properties. In contrast, biological methods (germination and fermentation) generally have a positive effect on aroma and typically lower pasting viscosity. Interestingly, the application of innovative nonthermal technologies is understudied in pulse flour matrices, although substantial research has been carried out on the main isolated components (starches, proteins, and fibers). The basic working principles of each technology and the available studies applying them to pulses are discussed, focusing on both static and dynamic high‐pressure processing, ultrasound, cold plasma, and pulsed electric fields. The selection of the optimal functionalization approach must be based not only on an understanding of the underlying mechanism but also on a clear view of the intended final application to ensure the desired ingredient performance and economic feasibility.

## 1 Introduction

Legumes, and specifically pulses such as beans, peas, and chickpeas, have been part of the human diet for centuries, serving as a valuable energy source with significant amounts of protein and fiber that remain extremely important for many people today, particularly in less developed countries. In recent years, increased demand for products with added protein, gluten‐free, and plant‐based options has also driven up the consumption and use of pulse flour in more developed countries, where intake still falls short of nutritional guidelines for pulses (Abu‐Ghannam and Gowen 2021; Asif et al. 2013; Hall et al. 2017; Sadohara et al. 2026).

In all cases where pulses are used as an ingredient, especially as the main one, functional and rheological properties are highly important, as they determine the performance of the mixture and the final product. Such broad industrial application requires not only standardized and reliable metrics to assess ingredient quality, but also the development of precise strategies to address specific issues and tailor properties to facilitate product utilization (Thakur et al. 2019). Common issues related to the techno‐functional properties of pulse flour include relatively low‐protein solubility, lack of viscosity improvement before heating, and pronounced retrogradation, among others. Additionally, beyond these technological issues, concerns also arise from a sensory perspective, as the flour often has off‐flavors such as beany and grassy notes, as well as nutritional concerns, due to the presence of antinutritional factors like phytic acid and raffinose oligosaccharides (Kaur and Majumder 2025; Keskin et al. 2022; Patterson et al. 2017).

To address these limitations, functionalization strategies can be used to induce specific changes in particle size, molecular structure, and component interactions, resulting in improved and predictable techno‐functional behaviors (Akharume et al. 2021; Hoover et al. 2010; Nikbakht Nasrabadi et al. 2021). These modification strategies can be grouped into three main categories based on their mechanisms of action: (i) chemical, which involves using specific reagents that bind to targeted flour components; (ii) biological, which involves using enzymes, either through exogenous addition or via fermentation and germination; (iii) physical methods involve applying heat (thermal treatments) or other inputs (such as pressure, electric fields, and so on) to induce structural and molecular modifications.

While the main strategies applied so far to pulse flours have been thermal and biological, research and the industrial market are now much more advanced in the functionalization of selected flour components, such as starch, proteins, and fibers. In this sector, many studies assess not only thermal and biological treatments but also focus on chemical modifications and nonthermal treatments (Ren et al. 2021; Zha et al. 2021). This different development in both the market and scientific research between the modification of flours and their isolated components may be due to factors such as higher economic value, which justifies the costly steps of functionalization, greater product homogeneity, which eases the modification steps, and a more specific set of functional properties that favor targeted applications and market needs.

Among all the mentioned techniques, innovative technologies are particularly understudied regarding their application to pulse flours, especially when the main aim is to assess effects on functional and rheological properties. Innovative technologies, often defined as nonthermal because their main mechanism of action is not primarily based on heat, include high‐pressure processing (HPP; both static and dynamic), ultrasound (US), pulsed electric field (PEF), and cold plasma (CP). Although initial interest in the food sector typically focused on their potential for microbial inactivation, subsequent research has expanded their possible use to many other areas of food science (Jadhav et al. 2021; Z.‐H. Zhang et al. 2019).

Regarding biomacromolecule modification, static high pressures have been found to affect starch by inducing loss of grain structural order and subsequent partial gelatinization, while the secondary and tertiary structures of proteins can be deformed in way that is not fully recoverable upon pressurization (Levy et al. 2021). US, and similarly dynamic high pressures, in contrast, affect these components through cavitation and high shearing forces, which can induce partial protein denaturation, particle size reduction, and starch granule damage (Guo et al. 2020; Qadeer et al. 2025; Wang et al. 2020). PEFs are thought to act through a combination of dipole charging and electrochemical reactions, which are plausibly capable of altering macromolecule structure (Giteru et al. 2018). CP, meanwhile, is based on the production of numerous ions, reactive species, and free electrons that can etch starch granules and induce polymerization or depolymerization of both starch and protein molecules (Basak and Annapure 2022).

Although the potential of innovative nonthermal technologies to alter functional and rheological properties has been assessed on isolated flour components such as proteins, starches, and fibers (with, in most cases, a good understanding of the mechanisms of action on starch and protein molecular and supramolecular structures) much less is known about their application in more complex matrices, such as pulse flour, where possible interactions between components might significantly change the expected outcomes.

This work briefly assesses the production, composition, and main characteristics of pulses and their flours. This is followed by a detailed review of conventional and innovative techniques commonly or recently applied to improve and modify pulse flour functionality. The review will focus on the advantages and disadvantages of each technique, with particular attention to the rheological and functional aspects of the flours. When information regarding pulses is limited, soy will also be considered.

## 2 Pulse Characteristics and Uses

### 2.1 Main Pulse Production Data

The Food and Agriculture Organization (FAO) uses the term legumes to include all crops belonging to the *Leguminosae* family. Within this large group, three main categories are identified: pulses (such as beans, chickpeas, and dry peas), oil‐bearing crops (including soy and peanuts), and vegetables (green peas and green beans). In 2020, among these crops, soy was the most produced worldwide (365 million tonnes), followed by peanuts (54 million tonnes), while all pulses together reached 91 million tonnse (with beans, chickpeas, and peas accounting for 27, 15, and 14.6 million tonnes, respectively). For comparison, in the same year, all cereals reached a production of around 3 billion tonnes (with maize, rice, and wheat being the most produced), a figure approximately 10 times the production of soy, which alone is four times the global production of pulses (FAOSTAT 2025). Beyond their global production relevance, pulses also play a crucial role in food security, particularly in drought‐prone regions of developing countries. Recent studies conducted in Sub‐Saharan Africa highlighted that the adoption of climate‐smart agriculture practices, such as crop diversification, soil conservation, and minimum tillage, can significantly improve the productivity of drought‐tolerant pulses including cowpea, pigeon pea, and green gram, thereby contributing to household food security and sustainable agricultural systems (Meti Kioko et al. 2024).

**FIGURE 1:**
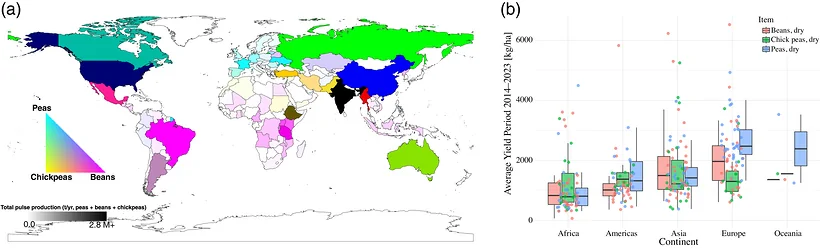
Production (a) and yields (b) for the three main pulse crops (beans, peas, and chickpeas) over the last decade (2014–2023). Personal elaboration from FAOSTAT data. Pink regions, such as Africa and South America, are characterized by higher bean production; yellowish areas, such as the Middle East, by chickpeas; and cyan areas, like Europe, by higher pea production.

Focusing on pulses, global production more than doubled from about 40 million tonnes in 1961 to 94 million tonnes in 2023, with India as the leading producer by far (shown in black in Figure 1a), followed by Canada, China, Nigeria, and Myanmar. Pulses yield also improved over the years, although yields vary significantly across regions and crops (Figure 1b). For example, in 2020, according to FAO data, overall pulses yield ranged from 772 kg/ha in Africa to 2020 kg/ha in North America, while global yields for peas, chickpeas, and beans were 2035, 1113, and 785 kg/ha, respectively. Although India produces about three times more pulses than the second‐largest producer, the types of pulses grown differ among countries, with Canada and Russia being main producers of peas, India and Brazil as the main producers of beans, and chickpeas produced predominantly in India and Turkey (Figure 1a; FAOSTAT 2025).

### 2.2 Seed Structure and Its Implications

Pulse seeds can vary widely in size and weight, with 100‐seed weights ranging from 0.5–5 g for lentils to 17–70 g for faba beans, as well as in shape (flat for lupins, spherical for peas) and color, but they share key characteristics (Ron 2015). All seeds are dicotyledonous, displaying a common structure that includes an external seed coat protecting the inner part of the seed, two cotyledons serving as energy storage for the future plant, and an embryo from which the new shoot will develop (Ron 2015).

**FIGURE 2:**
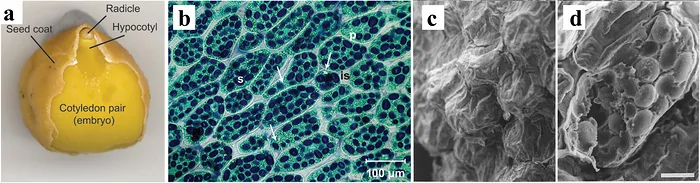
Main structure of chickpea seeds (a) and cellular organization in the cotyledons (b) from Wood et al. (2011). *p*: protein bodies; *s*: starch granules; *is*: intracellular spaces; *cw*: cell walls. (c) and (d) show the cell arrangement with starch granules and protein bodies from Marconi et al. (2000). Bar represents 20 µm. Images reprinted with permission.

The two cotyledons together account for 90% of the total seed weight and consist of specialized parenchyma cells that store large amounts of starch and proteins. These cells are tightly packed and held together by the middle lamella, a rigid structure composed mostly of pectin that is in direct contact with the cell walls. Inside each cell, starch grains are the main components, with sizes varying according to the pulse variety, but in all cases, the starch is embedded in a protein matrix made up of smaller protein bodies, primarily globular proteins (Figure 2; Marconi et al. 2000; Pallares Pallares et al. 2021).

The seed coat generally comprises 8%–16% of the seed weight, with thickness varying greatly between species (30–40 µm for lentils, 423 µm for chickpeas). This structure consists of multiple cell layers visible in the cross section, with characteristically higher amounts of cellulose and lignin in the cell walls, making the seed coat more stiff, resistant, and water repellent, all useful characteristics for a protective layer (Hughes and Swanson 1986; Marconi et al. 2000; Pallares Pallares et al. 2021; Zhong et al. 2018).

Seed microstructure and macrostructure are important and influence many aspects of pulse processing and applications. For example, the presence of a seed coat requires milling companies to use dehulling processes to remove the seed coat, often preceded by hydration to facilitate removal. This produces flour with lower fiber content and increased particle size homogeneity (Wood and Malcolmson 2021). Additionally, the dicotyledonous nature of the seeds allows for the production of split seeds (*dhal*, extremely common in Asia), with some pulses being more prone to splitting (lentils) than others (beans). Split seeds are smaller and have a higher surface‐to‐volume ratio, resulting in shorter cooking times (Asif et al. 2013). Differences in the ease of splitting and milling can also arise from the exomorphic microstructural features of the cotyledon's external layer (Jeganathan et al. 2022).

Obtaining flours with intact cotyledon cells, typically achieved through processes that break down pectins in the middle lamella (such as heating, or acid/alkali treatment), allows starches and proteins to remain bioencapsulated within the cell wall (Pallares Pallares et al. 2021). These processes lead to incomplete starch gelatinization and lower nutrient bioaccessibility (Ajala et al. 2023; M. Ma et al. 2017), characteristics that can be tailored for specific food products with a low glycemic index but can also significantly affect the textural and functional properties of the resulting products (e.g., lower thickening properties for intact chickpea cell flour compared to broken cells; Noordraven et al. 2021).

### 2.3 Flour Production and Milling Techniques

Seed milling is an established process with three main aims: (i) reducing particle size to obtain flour suitable for various purposes; (ii) separating unwanted components, such as the seed coat in pulses or the bran in cereals; (iii) tailoring specific component properties by controlling the degree of starch damage and the breakage level of cotyledon cells. Specifically for pulses, the milling step forms the basis for dry component separation processes (such as air classification, sieving, and electrostatic separation), which are gaining increasing traction in research (Scanlon et al. 2018; Vishwakarma et al. 2018).

Nevertheless, cereal and pulse milling differ significantly, requiring specific setup and optimization to achieve product quality and consistency. This is reflected in the lack of a formal definition for pulse flour, particularly regarding particle size, which is well defined for wheat (Thakur et al. 2019; Wood and Malcolmson 2021).

For pulses, milling typically begins with dehulling, which aims to properly separate the seed coat, followed by splitting the cotyledon, a characteristic that can be either positive or negative, depending on the intended use. Dehulling is usually performed with an abrasive mill. For peas, the milling operations are well studied and optimized, while for other pulses, the process remains more challenging due to specific shapes or a very adherent seed coat that requires pretreatment to improve dehulling efficiency. Such pretreatments include soaking and subsequent drying using weak basic solutions, or dry methods such as pitting, scratching, or sonication (Scanlon et al. 2018; Wood and Malcolmson 2021). Flour is produced from dehulled seeds using impact mills (e.g., pin, hammer, or centrifugal mills), which tend to produce fine particles with minimal starch damage and, if properly optimized, can efficiently separate starch granules from the protein bodies, resulting in a bimodal particle size distribution due to the different sizes of these components (Thakur et al. 2019; Vishwakarma et al. 2018).

As previously mentioned, interest in pulse milling processes focuses on fractionation and protein isolation. When dry milling is used, the subsequent steps involve a combination of three unit operations in varying order and repetitions: sieving, air classification, and electrostatic separation (Fernando 2021). These techniques separate the starchy and proteinaceous fractions by size (starch around 20–60 µm, protein > 10 µm), density (protein‐rich particles tend to be denser compared to starch‐rich ones) and electrostatic changes (protein‐rich particles can become much more electrically charged than starch during triboelectrification processes; Fernando 2021; Pulivarthi et al. 2023; Schutyser et al. 2025).

In contrast, when protein and starch isolation is performed using the traditional wet method (starch sedimentation and protein solubilization/precipitation), milling can also be done on soaked seeds although often the starting material for isolation is milled flour. The advantage of wet‐milling approach is that the materials are much softer to mill. This technique is commonly used for corn fractionation and starch extraction (Espinosa‐Ramírez and Serna‐Saldívar 2019; Wronkowska 2016).

### 2.4 Current Industrial Applications

Pulses have long been used in various ways by different cultures thus, resulting in traditional and well‐established applications that differ significantly across geographical regions. However, modern applications increasingly use pulse flours, as they require no soaking before cooking, making them easier to use. Most well‐studied applications are in the bakery sector, where pulse flour is often combined with wheat flour (WF) to improve nutritional value by increasing protein, fiber, and mineral content (Bravo‐Núñez and Gómez 2023; Hall 2021). WF substitution typically reaches up to 20%, since higher inclusion of pulse flours tends to negatively affect dough rheological properties, loaf texture, and gas retention. These issues stem from the absence of structuring components such as gluten in legume flours (Asif et al. 2013; Sozer et al. 2017). Additionally, such inclusion affects color and aroma (introducing beany and grassy smells) in the final products, making pulse flour easier to use in products where dough strength and rheological properties are less critical and other ingredients are present, such as crackers, biscuits, and cakes, compared to bread (Hall 2021).

Another major area of study regarding pulse flours application is the production of gluten‐free products. Legumes offer the advantage of being a good source of nutrients and can partially substitute other commonly used ingredients, such as starches from tubers or specific cereals (Hall 2021; Melini et al. 2017). Several studies have found that the rheological properties of batters used for gluten‐free products can be significantly improved with the addition of pulse flours, resulting in better microstructure, improved volume, and overall product acceptability (Melini et al. 2017; Sozer et al. 2017). This improvement is possible because the starting batter lacks gluten, so the fibers and proteins from pulses enhance water absorption, and increase batter viscosity (Herrera and Gonzalez de Mejia 2021).

Other applications for pulse flours include snacks, such as bars or chips, where extrusion technologies can create new textures. The use of pulse flour as the main ingredient, either alone or combined with cereals, is being thoroughly explored. In these products, extrusion can be the sole process ensuring structure and product stabilization, thanks to the high temperature and shearing forces applied to the batter. However, frying or baking is also often combined with extrusion to achieve the desired product characteristics (Escobedo and Mojica 2021; Sharma et al. 2022).

Industries and researchers are increasingly using not only whole pulse flour but also specific fractions, such as starches, proteins, and fibers with varying degrees of purity. These products offer properties that are markedly different from those of the original flour and can be tailored for each application (Boye et al. 2010; Shevkani et al. 2019). Generally, protein fractions exhibit higher emulsifying and foaming capacities (FCs) due to their high content of amphipathic components, while starch‐rich fractions can provide much greater thickening potential upon thermal treatment because of starch gelatinization. Typical applications for protein isolates (PIs) or protein concentrates (PCs) include use as meat extenders in sausages or patties, improving water retention and reducing cooking losses (Rivera et al. 2024). In pasta production, pulse protein fractions have been shown to enhance the nutritional profile and increase pasta firmness after cooking, although they also result in higher cooking losses. The main application for pulse PIs and PCs is the production of “*meat alternative*” products, high‐protein products designed to resemble some of the nutritional, functional, and sensory properties of traditional animal‐based products (Hopf et al. 2023; Riaz 2020). In these products, pulse proteins are the primary ingredient, combined with starches and specific thickeners to achieve the desired functional and nutritional properties. The key differences between PIs and PCs lie not only in their degree of purity but also in the isolation process, with PIs generally obtained through harsher isolation methods, which can also affect the protein state and worsen their functional properties.

Pulse starches can be used as thickeners in various products, with gluten‐free foods being a major application. They are also important binders for extruded snack bars (N. Kumar et al. 2025; Ren et al. 2021; D.‐T. Wu, Li, et al. 2023). Similarly, although pulse fibers are primarily used as functional ingredient to increase fiber content in food products, they are also useful for controlling batter rheology and as a filler in meat products (N. Kumar et al. 2025; Tosh and Yada 2010; D.‐T. Wu, Li, et al. 2023).

Sadohara et al. (2025) reported that from 2012 to 2021, there was a 4.1‐fold increase in the launch of new food products containing at least one pulse ingredient in the US market, demonstrating steady growth in consumer interest. This demand has been driven by key factors, including the perception of pulses as a healthier option due to increased protein and fiber content, as well as expansion in market niches such as vegan/vegetarian, gluten‐free, and nonallergenic products (Lascialfari et al. 2019; Sadohara et al. 2022). Nevertheless, hurdles remain that slow the adoption of pulse‐based products, including low sensory acceptability, high prices, and, from an industrial perspective, high variability in flour specification between producers (low ingredient standardization; Lascialfari et al. 2019; Sadohara et al. 2022).

## 3 Functionalization Techniques for Pulse and Legume Flours

Pulse flours (e.g., pea, faba, lentil, chickpea, bean) are increasingly used to boost protein content, replace allergens and gluten, and support clean‐label, plant‐based formulations. However, native flours often have beany off‐flavors, inconsistent hydration, antinutritional factors, and lack of structuring agents such as gluten. Functionalization by tailoring molecular and supramolecular structure to achieve targeted techno‐functional behaviors can address these gaps.

**FIGURE 3:**
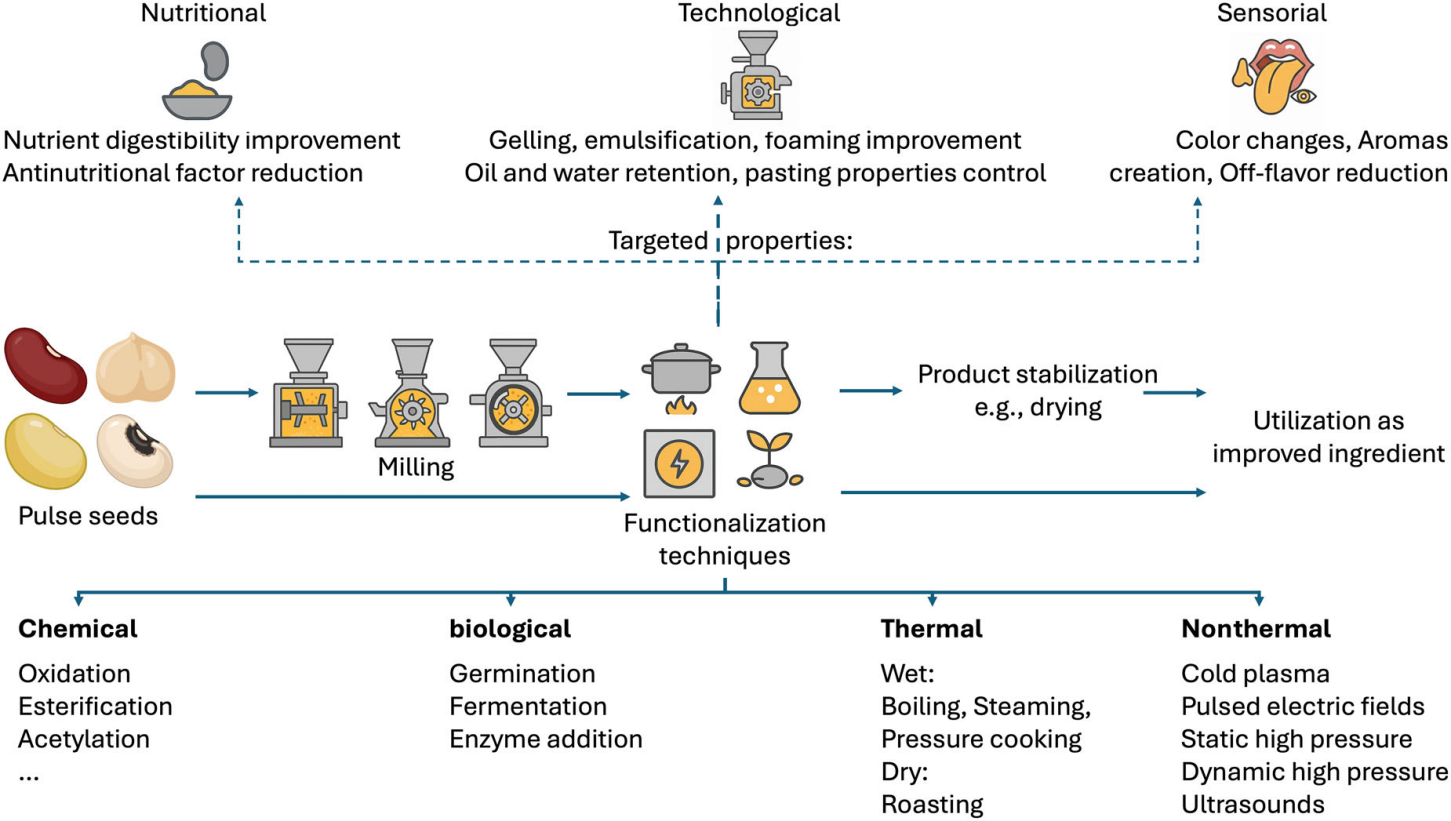
Schematic representation of the steps that can be performed to optimize the main targeted properties in pulse flour, namely, nutritional, technological, and sensorial.

Functionalization methods vary widely and are generally categorized as physical (thermal and nonthermal), chemical, and biological strategies (Figure 3). Physical methods broadly include milling procedures (to control particle size and starch damage), air classification (to adjust the starch–protein ratio), thermal treatments (wet and dry, to modulate starch gelatinization and protein denaturation), and novel nonthermal techniques such as HPP, ultrasonication, PEF, and CP. Biological approaches, such as germination, fermentation, or the use of specific enzymes (proteases, amylases, transglutaminase), offer alternative routes to similar results. Chemical methods, including acetylation, deamidation, or crosslinking, represent another option for flour functionalization.

Functional properties are critical when flour is used, and their control and optimization must be guided by the intended purpose and application. Key evaluated properties include hydration and bulk behaviors, measured by water‐holding capacity (WHC) and oil‐holding capacity (OHC), swelling power, and solubility. Pasting profiles (viscosity changes during simulated cooking) and thermal transitions (DSC enthalpy/temperature) quantify starch–protein modification as a function of temperature. Rotational and oscillatory rheology, combined with gel strength textural assessment, describe gelation and formability in doughs, batters, and meat analog matrices. Interfacial performances are assessed by emulsifying activity and stability indices (EAI/ESI), creaming stability, and foamability or foam stability. Collectively, these parameters provide a useful framework for designing pulse flours with predictable functionality across beverages, baked goods, emulsions, and structured plant‐based foods.

### 3.1 Thermal Methods

Thermal treatments are among the most extensively studied techniques for pulse flour functionalization, with some of the earliest research dating back to the 1980s. The main advantage of thermal treatment is its wide range of possible applications, as heat can be applied to whole seeds or flours, under dry or wet conditions, using conventional methods (such as boiling or roasting) or innovative heating techniques (such as microwave heating, infrared irradiation, or radiofrequency heating). Generally, more studies focus on whole seeds before flour production, with objectives including not only flour functionalization but also aroma improvement (reduction of beany aroma), reduction of antinutritional factors (such as deactivation of trypsin inhibitors), and color optimization (whitening, which is generally preferred for bread‐making applications; Y. Kumar et al. 2022; Roland et al. 2017).

Table 1 summarizes literature findings on the effect of thermal treatments on the functional properties of pulse flours. WHC (or water adsorption capacity) is typically improved, though the extent varies greatly (from a 200% increase reported by Ferawati et al. 2019, to a 50% increase observed by Asavajaru et al. 2025 both for yellow peas). Similar improving trends have been noted for OHC, though generally to a lesser degree than WHC, with maximum increases reported around 19%–38% (Choe et al. 2022; Guldiken et al. 2022; Laing et al. 2023; Onimawo and Akpojovwo 2006; Stone et al. 2021), while some studies have also reported decreases (Asavajaru et al. 2025; Bento et al. 2022). The stronger impact of heating on WHC compared to OHC is consistent across studies and likely results from the effect of heating, especially in moist environments, on starch granules. In fact, the induction of gelatinization disrupts the organized molecular structure inside the grains, allowing amylose and amylopectin to better interact with water through hydrogen bonding. In contrast, OHC is often linked to the proteins present in the flour rather than the starch fraction. Moreover, after treatment, foaming and emulsifying properties consistently decrease across studies, with reductions ranging from −15% to −50% (Asavajaru et al. 2025; Ferawati et al. 2019; Laing et al. 2023; Onimawo and Akpojovwo 2006) to complete loss of FC (Guldiken et al. 2022). For these properties, available studies tend to link them to the protein fraction, which upon treatment may undergo denaturation and aggregation, leading to the partial loss of available lipophilic sites that can bind either to fats or air bubbles (Ferawati et al. 2019; Z. Ma et al. 2011).

**TABLE 1:** Effect of thermal treatment on pulse flours functional properties.

Form—Pulse type	Treatment type	Key results	References
Flour—Lentil, chickpea, and pea	Roasting (1 min, 80°C), boiling (90°C, 20 min)	Boiling: ↑ WHC, OHC and gelling; ↓ solubility and ANFs Roasted flour presented similar functional properties to control but lower ANFs	Z. Ma et al. (2011)
Flour—Navy bean	Roasting (15 min, 120–190°C), extrusion (130°C, 22%–30% RH, 250–500 rpm)	Dry heat: ↑ protein denaturation, pasting properties and b*; ↓ WSI, a*. Extrusion: ↑ particle size, protein denaturation, *G*′ and b*; ↓ starch crystallinity, pasting properties.	Navneet et al. (2024)
Flour—Three beans and chickpeas	Jet cooking (138°C) followed by drum drying (10% solids)	↑ WAI, probiotic sugars availability, soluble fibers; ↓ WSI, pasting viscosity, particle size	Felker et al. (2018)
Seed—Pinto, navy, kidney, and black beans	Roasting (165°C, 20 min), boiling (100°C, 90 min)	Wet treatment: ↑ WHC, digestibility and particle size; ↓ viscosity Dry treatment: ↑ OHC and aroma; ↓ viscosity and particle size	Choe et al. (2022)
Seed—Yellow pea	Radio frequency (85 or 115°C final temperature, 14% RH)	↑ WHC and LGC; ↓ ANFs, OHC, FC and emulsifying properties. Higher temperature induced more pronounced changes.	Asavajaru et al. (2025)
Seed—Four beans variety	Steaming dry beans (20 min, 121°C) steaming soaked beans (5 min, 121°C)	OHC unchanged; ↑ pasting temperature; ↓ WSI and pasting viscosity. Δ*H* always lower for soaked beans compared to unsoaked	Bento et al. (2021)
Seed—Six beans variety	Steaming upon seed soaking (5 min, 121°C)	↑ Pasting temperature and WAI; ↓ pasting viscosity, gel hardness, Δ*H* and WSI. OHC and emulsifying properties have been affected in a variety dependent manner	Bento et al. (2022)
Seed—Faba, white beans, yellow, and gray peas	Roasting (15–25 min, 180°C), boiling (30–50 min) and germination	OHC and WHC increased in all treatments, with roasting being the best preforming. FC and FS lowered by all treatments compared to control	Ferawati et al. (2019)
Seed—Navy bean, chickpea	Infrared heating (120–140°C, 20%–30% RH)	↑ WHC, OHC, starch digestibility and PDCAAS score; ↓ emulsion capacity, FC and solubility. Pasting viscosity was increased for navy beans and decreased for chickpeas	Guldiken et al. (2022)
Seed—Chickpea, lentil, pea, navy bean	Roasting (30 min, 160°C, 20%–30% RH)	↑ WHO, OHC and starch digestibility; ↓ α‐amylase activity, FC and solubility. Emulsion capacity and PDCAAS did not change significantly	Stone et al. (2021)
Seed—Pigeon pea	Roasting (60 min, 80–100°C)	↑ WHC and OHC; ↓ foaming, emulsifying properties and ANFs	Onimawo and Akpojovwo (2006)
Seed—Peas, lentil	Infrared heating (120–140°C, 20%–30% RH)	↑ Damaged starch, WHC and OHC; ↓ foaming and emulsifying properties, solubility; (overall, minor functional modifications)	Laing et al. (2023)
*Studies assessing food applications*			
Flour—Chickpea, lentil mung bean and black gram	Pressure cooking 10 min	↑ WHC; ↓ protein solubility at all pH and FC. Lower overall acceptability of deep‐fried products produced	Nagmani and Prakash (1997)
Flour—Yellow peas	Microwave (20 min, 10% power), roasting (3 h, 130°C)	Marginal viscosity increments. When used to make steamed bread, both treatment reduced negative impact of wheat substitution (↑ specific volume; ↓ hardness)	Sun et al. (2023)
Flour—Navy and pinto beans	Extrusion (85°C, 36% RH)	↑ WSI and WAI; ↓ pasting viscosity. Improved overall sensory acceptability for extruded flour. Control sample was commercially steamed (82°C) flour.	Siddiq et al. (2013)
Seed—Yellow peas	Conventional or Revtech roasting (21–30 min, 120–160°C)	Both treatments induced ↓ specific volume and crumb firmness compared to control breads, with 120°C being the most effective temperature.	Young et al. (2020)
Seed—Yellow peas	Heating (5 min, 120–160°C) with or without steam (15%)	When used to make bread, ↓ off‐flavor and specific volume for all tested sample, while treated flours reduced negative impact of wheat substitution (↑ overall acceptability) for split peas but not for whole peas.	Sopiwnyk et al. (2020)

Abbreviations: ANFs, antinutritional factors; FC, foam capacity; FS, foam stability; LGC, last gelation concentration; OHC, oil‐holding capacity; PDCAAS, protein digestibility corrected amino acid score; WAI, water adsorption index; WHC, water‐holding capacity; WSI, water solubility index.

Unless otherwise stated all comparison are done against untreated flour as control.

As shown, although general trends are consistent across studies, the magnitude of the impact can vary greatly. While this can be explained by inconsistency in the treatment parameters used (final temperature, cooking time, moisture used) and in assessment methodology, studies that observe the impact of the same treatments on different pulses highlight that botanical origin can also play a role. Stone et al. (2021) observed that chickpeas lost 94% of their foam capacity upon roasting, compared to an 8% gain observed for lentils. The authors attribute such a marked difference not only to varying initial composition but also to different protein fraction behavior; while solubility markedly decreased in chickpeas, lentil flour retained part of its protein solubility, thus allowing for better foam stabilization. Ferawati et al. (2019) observed that while yellow peas tripled WHC upon roasting, white beans only increased by 80%. Concomitantly resistant starch increased markedly in peas but decreased in beans, suggesting that different types of starch crystals and amylose‐amylopectin ratios can play a role. Nevertheless, a study explicitly investigating the reasons behind the different pulse responses to heat treatment, taking into consideration both composition and microstructure, is needed to properly understand these phenomena and optimize treatments.

A notable difference within heat treatments is the presence of moisture. Wet systems, such as boiling or cooking after soaking, allow faster and more efficient heat transfer, though they limit the final temperature to 100°C (the boiling point of water) unless pressure cookers are used. The amount and availability of water also critically affect macromolecules such as starch, where gelatinization is strongly inhibited at low moisture contents, or pectins, where higher moisture contents enable faster pectin softening through demethylation and solubilization. Choe et al. (2022) compared dry‐ and wet‐heat treatments on different whole beans (black, kidney, navy, and pinto), observing that moist‐heat treatments increased WHC (a 46%–79% increase compared to control) more effectively than dry‐heat treatments (from −5% to +10% compared to control), with a similar trend observed for starch and protein digestibility. In contrast, OHC was affected more by dry‐heat treatments (a 13%–26% increase compared to control) than by boiling (from −11% to +3% compared to control). Moreover, both treatments decreased pasting properties; for example, final viscosity was reduced by 96%–98% by dry treatments compared to control, while wet treatments reduced final viscosity by only 43%–80%. When comparing wet‐ and dry‐heat treatment on flours (not seeds), Z. Ma et al. (2011) observed that wet‐treated flours showed improved WHC and OHC, reduced protein solubility, and no visible starch grains in SEM, while roasted flour exhibited similar functional properties and structure to the control. Their work highlighted the difference between performing wet treatment on whole seeds and flours. In the case of flours, all components can properly hydrate, water diffusion is not a limiting factor, and starch granules can fully gelatinize and dissolve, forming a network that entraps protein and other particles. When wet treatment is applied to whole seeds, gelatinization occurs only to a much more limited extent and is constrained within the cell walls, hindering the formation of a gel network. Another confirmation of this behavior was reported by Bento et al. (2021), where soaked steamed beans consistently showed lower gelatinization enthalpies compared to dry steamed beans, even though the steaming time was four times longer for dry seeds (20 min instead of 5 min).

More recently, dry heating was also compared to extrusion processes for navy bean flour. Navneet et al. (2024) observed that dry‐heated flour retained starch grain integrity but showed signs of protein bodies denaturation (assessed by FTIR). When mixed with water, the flour exhibited faster grain swelling and overall improved pasting properties. In contrast, the high shearing forces of extrusion induced starch grain disruption and the release of amylose and amylopectin, along with marked protein denaturation. Although these impacts resulted in lower pasting properties, the flour could form gels at room temperature, with a *G*′ values of approximately 4000 Pa compared to approximately 4 Pa for the control.

Finally, studies have also assessed the applicability of thermally treated pulse flour in producing foods, specifically bakery goods. The general pattern that emerges is that substituting wheat with pulse flour inevitably results in reduced acceptability of the product due to lower bread volume, harder structure, and unpleasant aromas. Nevertheless, when heat‐treated flours are used, the negative impact of WF substitution is reduced, resulting in better sensory acceptability and improved loaf texture, volume, and hardness (Siddiq et al. 2013; Young et al. 2020). Sun et al. (2023) observed that the 25% disliking of steam bread with partial WF substitution was improved to only 8% disliking using microwaved pea flour. Similarly, Sopiwnyk et al. (2020) found that the increase in bread hardness with WF substitution (reaching 346 g from 219 g) was reduced to only 302 g using thermally treated whole pea flour.

### 3.2 Biological Methods

Among the functionalization alternatives typically considered “biological” are germination, fermentation, and the use of enzymes. Interestingly, in the first two cases, the flour composition is actively changed, either due to nutrient consumption by microorganisms (MOs) or by the embryo developing into root and shoot. Such a marked compositional shift does not occur in other modification techniques, where structure, conformation, or interaction between components are mostly altered.

Germination is generally divided into three phases. First, the seed is soaked, allowing water diffusion and seed coat softening. Second, water absorption slows, and the metabolic activity of the embryo restarts, with the production of enzymes (α‐amylase and proteases) enabling the breakdown of starches and proteins for shoot growth. Finally, there is a second increase in seed imbibition, allowing easier nutrient movement toward the developing embryo. In general, germination ends with the emergence of the radicle, at which point seeds are dried to halt the reaction and allow for seed milling (Avezum et al. 2023; Onwuka et al. 2024).

After germination, pulse flour tends to show a looser cotyledon structure, possibly due to water penetration and enzymatic effects on cell walls and the middle lamella, although starch grains still seem to be relatively unaffected (Mao et al. 2024; Setia et al. 2019). This may be important, as it could impact the milling process.

Table 2 reports literature results regarding the effect of germination on functional properties of pulse flours. Most studies highlight an improvement in WHC (from 9% to 130%) and FC (from 14% to 60%) proportional to the germination time, while reports on OHC (from 17% reduction to 28% increment) and emulsifying activity are more contradictory. There is, however, general agreement on a lower gelatinization enthalpy, suggesting that partial loss of granule crystallinity occurs during germination. Nevertheless, it is important to note that the high variability in the effect of germination effect is reported among pulse species and varieties, as observed by Ghavidel and Prakash (2006) and Mao et al. (2024), with lentil increasing in WHC by 50%, while chickpeas only 11%. Unfortunately, for the measurement of functional properties, the methodologies used tend to differ slightly between studies, making comparisons more meaningful when only trends are considered rather than exact values. A solution comes from the work of Ghavidel and Prakash (2006) who compared four different pulses using a consistent methodology. Interestingly, while emulsification capacity was improved relatively uniformly between pulses (27%–35% increase), the same was not true for water and fat absorption capacities, where improvements ranged from 11% to 48% and 6%–25%, respectively. The authors do not identify a specific reason for this different behavior, but they link it to the varying germination rates and enzymatic mechanisms activated in each pulse during germination. In a more recent study comparing yellow peas and faba beans, the authors suggest that, in addition to different germination rates between the two species, the different behavior may also arise differences in composition and seed microstructure. Specifically, faba beans have higher protein and fiber content, resulting in more compact microstructures that completely entrap starch granules, while peas present starch granules already visible in ungerminated samples. Thus, the greater loosening of the matrix upon germination allows faba bean flour to significantly improve some functional properties compared to peas (Setia et al. 2019).

**TABLE 2:** Studies assessing germination treatment on pulse flours.

Form—Pulse type	Treatment type	Key results	References
Seed—Grass pea	Germination (48 h, 25°C)	↑ WHC, OHC, FC and EC; ↓ pasting viscosity and Δ*H*	Lakshmipathy et al. (2024)
Seed—Pigeon pea	Germination (24, 48, and 72 h, 28°C)	↑ WHC, FC and EC; ↓ OHC, FS, LCG and Δ*H*. Pasting viscosity improved after 24 h then reduced	Chinma et al. (2022)
Seed—Yellow pea, faba bean	Germination (24, 48, and 72 h, room temperature)	↑ WHC, FC and EC; ↓ Δ*H* and FS. Pasting viscosity improved after 24 h then reduced	Setia et al. (2019)
Seed—Lentil, chickpea, cowpea, and bean	Germination (24 h)	↑ WHC, OHC, EC and FC; ↓ FS	Ghavidel and Prakash (2006)
Seed—Chickpea	Germination (72 h, 25°C)	↑ WHC and FC; ↓ Δ*H* and FS; OHC and EC unaffected	Mao et al. (2024)
Seed—Chickpea	Germination (36 h, 30°C, 85% RH)	↑ WHC, OHC and gel syneresis; ↓ pasting viscosity, pasting temperature and Δ*H*	Sofi et al. (2023)
*Studies assessing food applications*			
Seed—Yellow pea, faba bean	Germination (72 h, room temperature)	↓ Pasting temperature, pasting viscosity. On bread formulation germination had detrimental effect on loaf volume and crumb hardness, albeit comparable to control pulse flour	Setia et al. (2020)
Seed—Chickpea	Germination (time not specified)	No significant improvement in functional properties. Lower moisture and hardness in cookies. Unchanged overall acceptability	Dogruer et al. (2023)

Abbreviations: EC, emulsion capacity; FC, foam capacity; FS, foam stability; LGC, last gelation concentration; OHC, oil‐holding capacity; WAI, water adsorption index; WSI, water solubility index; WHC, water‐holding capacity; Δ*H*, gelatinization enthalpy.

Unless otherwise stated all comparison are done against untreated flour as control.

Interestingly, studies on different pulses seem to agree that longer germination times (2–3 days) tend to reduce peak and final viscosities (from −18% up to −64% for final viscosity), whereas shorter germination (24 h) result in improved viscosities (final viscosity increased from 8% to 39%), while all pasting temperature tend to be reduced (Chinma et al. 2022; Lakshmipathy et al. 2024; Setia et al. 2019; Sofi et al. 2023). The reason behind the increase in pasting viscosity up to 24 h could be due to a loosening of the cotyledon structure and an increased detachment between starch and protein bodies, probably due to the enzymatic effect. This phenomenon can lead to better starch hydration and thus higher viscosities, while excessive enzymatic activity (> 24 h) can damage the starch grain excessively thus hindering the gelatinization process (Chinma et al. 2022; Setia et al. 2019). Finally, studies agree in highlighting generally improved starch (higher RDS) and protein digestibility (higher IVPDCAAS) thanks to the enzymatic effect of germination combined with lower ANFs (Avezum et al. 2023; López‐Martínez et al. 2017).

A wide range of MOs and fermentation methods have been tested so far. Table 3 presents recent studies evaluating the functionalization of pulse flour by fermentation. Generally, fermentation can be performed on either whole seeds or flour, but it always occurs after soaking and wetting the product. Depending on the degree of wetting, fermentation can be classified as solid‐state fermentation (SSF, typically performed with fungi and molds) or submerged fermentation (SF, usually used for lactic acid bacteria or yeasts). Another important aspect of fermentation processes is that the starting product often needs to be sterilized to eliminate initial microbial contamination. As a result, the flour undergoes specific changes, similar to those observed in wet thermal treatments (mainly starch gelatinization and protein denaturation), which also play an important role in the functional properties of the final products. Finally, fermentation processes are characterized by a relatively long duration (generally around 3–4 days, but up to 14 days in some studies), which can reduce their industrial appeal.

**TABLE 3:** Studies assessing fermentation treatment on pulse flours.

Form—Pulse type	Treatment type	Key results	References
Flour—Faba bean	SSF, *A. oryzae* (28°C, 48 h) or *Rz. oligosporus* (30°C, 72 h)	↑ WHC and particle size; ↓FC, solubility and LGC	Gautheron et al. (2024)
Broken seeds—Red bean	SSF, *Cordyceps militaris* (25°C, 7 days)	↑ WHC, OHC, and EC; FC unaltered	Xiao et al. (2018)
Seed—Chickpea	SSF, *Cordyceps militaris* (25°C, 7 days)	↑ WAI, WHC, OHC, EAI, and ESI; ↓ WSI	Xiao et al. (2015)
Seed—Pigeon pea	SSF, (30°C, 3 days) after germination	↑ WHC, OHC, and FC	Sobowale et al. (2024)
Flour—Backeyed pea	SSF, *A. oryzae* (30°C, 0–96 h)	↑ WHC, OHC, FC, FS, EC, and ES	Chawla et al. (2017)
Seed—Chickpea	SF, *L. Case*i and coculture *L. acidophillus Bifidobacterium* BB12 *S. termophilus* (28°C, 0–96 h)	Minimal functional changes recorded	Estanech et al. (2024)
Flour—Chickpea	SF, *L. plantarum* or *W. Paramesenteroides* (30–37°C, 8–24 h)	↑ Emulsion activity; ↓ LGC. WHC and OHC unaffected	Sáez et al. (2022)
*Studies assessing food applications*			
Flour—Kidney beans	SSF, *A. awamori* (30°C, 0–96 h)	↑ WHC, OHC, FC, FS, EC and ES; ↓ overall acceptability of biscuits prepared with wheat substitution greater to 50%	Patil et al. (2025)
Seeds—Kidney bean	SSF, *P. ostreatus* (25°C, 14 days)	↓ Bread hardness, lower acceptability	Espinosa Páez et al. (2023)
Seeds—Pigeon pea	SSF (24–48 h, temperature and MO unspecified)	↓ Lower pasting viscosity. Lower amylose present. Cookies overall acceptability decreased	Sobowale et al. (2025)
Seeds—Chickpea	SF, *Bifidobacterium animali* and *Lp. plantarum* (37°C, 24–48 h)	Loaf volume unaffected compared to control chickpea flour. Faster hardness increases during storage	Skrzypczak et al. (2024)
Flour—Lentil and chickpea	SF, *Lp. plantarum* and *Le. brevis* (30°C, 1–10 days)	Gelatinized fermented flour reduced hardness in bread and pasta	Montemurro et al. (2025)

Abbreviations: EAI, emulsion activity index; EC, emulsion capacity; ES, emulsion stability; ESI, emulsion stability index; FC, foam capacity; FS, foam stability; LGC, last gelation concentration; OHC, oil‐holding capacity; SF, submerged fermentation; SSF, solid‐state fermentation; WAI, water adsorption index; WHC, water‐holding capacity; WSI, water solubility index.

Unless otherwise stated all comparison are done against untreated flour as control.

Regarding the effects on flour properties, fermentation processes exert the greatest impact on color (Δ*E* reaching up to 25; Gautheron et al. 2024; Sobowale et al. 2025), aroma, and chemical composition of the final flour, due to the intense metabolic activity of the MO, along with the greatest variability in the end product because of the high number of MO that can be used for fermentation. Studies focusing on functional properties have shown that fermentation generally increases WHC (from 9% up to 80%) and OHC (from 9% up to 80%), a result that sometimes was attributed to higher production of soluble molecules and increased matrix porosity. FC and emulsion properties can also sometimes be improved (although high variability is reported between studies, with some showing up to 150% increases and others a 53% decrease), mainly explained by the production of amphipathic peptides after proteolytic effects (Chawla et al. 2017; Gautheron et al. 2024; Patil et al. 2025; Sáez et al. 2022; Xiao et al. 2015, 2018). Conversely, pasting viscosity is observed to decrease proportionally to fermentation time, possibly due to starch damage during fermentation and the subsequent consumption by MOs (Sobowale et al. 2024).

In a recent study, Montemurro et al. (2025) assessed the effect of lentil and chickpea (pregelatinized or not) sourdough type‐II fermentation on bread and pasta production. The results showed that the microbial population did not differ between raw and pregelatinized flour, although the amount of acetic acid was higher for pregelatinized flour after 1 day of fermentation. Regarding the bread properties, the authors observed opposite effects of fermentation on bread hardness. While unfermented pregelatinized lentil flour resulted in higher hardness compared to raw flour (71.0 vs. 38.6 kN/m^2^), fermentation led to an increase in hardness in bread made from raw flour, reaching 53.5 kN/m^2^ while causing a decrease in bread made from pregelatinized flour, up to 59.5 kN/m^2^. These results highlight the complex nature of changes occurring during fermentation, indicating the need for a better understanding of the process and its implications to optimize flour modification.

### 3.3 Chemical Methods

There is a lack of studies assessing the chemical modification of pulse flours, especially regarding the subsequent assessment of functional properties. This may be due to several factors, such as the generally low economic value of pulse flour, which may not be high enough to justify such treatment. Moreover, as discussed, there are already multiple ways to tailor functional properties in flours, making chemical modifications less attractive, since these techniques generally require the use of specific reagents that can leave trace amounts requiring specific labeling, which may discourage consumer acceptance. Finally, the high variability in composition and the presence of multiple components in the flours may make the application of such treatments more complex and inconsistent due to possible interferences.

Sinaki et al. (2021) assessed the effects of commonly used oxidizing agents (benzoyl peroxide, BP; azodicarbonamide, ADA; and oxygen from air) on pea flour during the extrusion process. They found that, at temperatures below 110°C, oxygen in air was the best oxidizing agent to improve solubility (from 15% up to 22.7%), while emulsion capacity and stability were greatly improved by BP and ADA (44% and 50%, respectively, compared to 39% and 37% for the control and air, respectively). Nevertheless, the authors had difficulty in pinpointing the exact mechanism behind the observed changes in functional properties. In another study, Araoye et al. (2024) performed acetylation on lima bean flour using acetic anhydride. Interestingly, acetylation improved OHC and WHC, with better results at a lower degree of acetylation (2.11%), while pasting viscosity was strongly increased regardless of acetylation degree.

In contrast to flours, there are numerous scientific publications assessing chemical modifications of specific pulse flour fractions, particularly starches and proteins. PIs can undergo deamidation (Chen et al. 2021), phosphorylation (Hu et al. 2023), glycosylation (Naik et al. 2022), and acetylation (Araoye et al. 2024). Similarly, starches can undergo acetylation, oxidation, crosslinking, and acid‐thinning. For a comprehensive summary and explanation of the effects and mechanisms of chemical modification in starches, see Bangar et al. (2022) and Ren et al. (2021).

## 4 Innovative Nonthermal Functionalization Techniques

Innovative functionalization techniques encompass a wide range of technologies with relatively limited industrial use and mechanisms of action not strictly reliant on thermal effects, although all such applications may cause some heating. Nonthermal technologies have not been directly investigated for improving flour properties. In fact, they were initially studied and applied in the food sector to enhance extraction and microbial safety. The following section presents an up‐to‐date overview of the literature evaluating the use of these technologies to modify pulse flour properties. When studies on pulses are not available or limited, the effects on other legumes (e.g., soy) are included. Nevertheless, due to the markedly higher oil content of soy seed and the subsequently different microstructure it is important to acknowledge that results obtained on soy might not be directly reproducible on pulse seeds.

### 4.1 Static High‐Pressure Processing

High hydrostatic pressure (HHP) is a processing technique with numerous applications in the food industry. It operates by applying static pressures of up to 1000 MPa to prepacked products placed in chambers that are filled with water and pressurized. During operation, the water transmits pressure instantaneously and uniformly to the products being treated. Under these conditions, any phenomenon, such as a chemical reaction or phase transition, accompanied by a decrease in volume is favored by an increase in pressure, according to Le Chatelier's principle, which states that at equilibrium, a system tends to minimize the effect of any external force applied. On macromolecules, these effects result in a change in their spatial conformation, leading to the eventual rupture of weak bonds (van der Waals forces, hydrogen bonds, and ionic interactions). For proteins, the deformation can induce loss of secondary and tertiary structure and even gelation, while in starch, the granule crystalline structure can be altered. It must be noted that the observed modification can often be only partially recovered upon pressure release. These effects underlie the observed modification of functional properties (Aganovic et al. 2021; Castro et al. 2020; W. Wang et al. 2022).

Many applications have focused on pulses, with most studies examining effects on flours, while only one has directly investigated seeds. Table 4 presents the main results obtained by applying HHP to modify pulse functional properties. When treatments were performed on whole seeds, the authors found that HHP‐induced effects were strongly dependent on the pulse type, with jack beans being the least affected and showing no statistically significant differences in OHC and ES after HHP treatment (Sosa et al. 2020). FC increased in cowpea (from 31% to 60%) after pressurization, while it decreased in pigeon pea (from 58% to 27%). The markedly different response of functional properties after HHP treatment among various botanical origins is extremely interesting, and the authors attribute it primarily to differences in protein content and composition. Specifically, while all beans had a main fraction around 45–67 kDa, jack beans presented a predominant band at 94 kDa. Interestingly, this protein band persisted under reducing conditions, indicating that nondisulfide interactions might make it structurally resistant to pressure. Nevertheless, the possible effect of different microstructures should not be overlooked, as it was observed that the presence of nonstarch particles adhering to starch granules had a protective effect on the starch itself upon pressurization (Z. Liu et al. 2021).

**TABLE 4:** Studies assessing HPP treatment on pulse flours.

Form—Pulse type	Treatment type	Key results	References
Flour—Chickpea	HHP (150–600 MPa, 15 min holding, 25°C, 16.6%–33% slurry)	↓ Gelatinization temperature and Δ*H*. Stronger effect recorded for lower concentrations. Before gelation pressure increased viscoelasticity while after gelation, viscosity decreased for higher pressures.	Alvarez et al. (2014)
Flour—Chickpea	HHP (200–600 MPa, 15–25 min holding, 10–50°C, 16.6% slurry or dry)	On treated powder ↓ gel hardness and cohesiveness with pressure more important than temperature or time. Highest effects recorded for slurries while minor effect observed on treated powders	Alvarez et al. (2015)
Flour—Chickpea	HHP (200–600 MPa, 15–25 min holding, 10–50°C, 16.6% slurry)	↓ *G*′ and *G*″; ↑ gelatinization temp. Treatment pressure main driver, followed by temperature and time.	Alvarez et al. (2016)
Flour—Pea and faba	HHP after autoclaving (350 MPa, 10 min, 23°C, 25% slurry)	↓ Pasting temperature; ↑ RS%, EA, and ES. Unaltered pasting viscosity, WSI, and WAI	Arroqui et al. (2024)
Flour—Chickpea, pea, soy	HHP (200–400 MPa, 10 min, 20°C, 50%–62.5% slurry)	↓ Pasting viscosity, protein solubility, and batter stickiness; ↑ Zero shear viscosity	Angioloni and Collar (2013)
Flour—Soy	HHP (200–600 MPa, 25–90°C, 1–60 min, 50% slurry)	↓ Δ*H*; ↑ To and Tp. Sucrose and betains solution work as baroprotectant against protein denaturation.	Kweon et al. (2017)
Flour—Pinto bean	HHP (150–600 MPa, 5–15 min, 22°C, 33% slurry)	↓ Δ*H* and protein solubility, WHC, EAI ↑To, Tp. Pasting viscosity increased only for 600 MPa.	Lin and Fernández‐Fraguas (2020)
Flour—Mung bean	HHP (300–600 MPa, 20 min, 25°C, 20% slurry)	↓ Crystallinity, particle size, To, ∆*H* and pasting viscosity; ↑ pasting temperature	Z. Liu et al. (2021)
Seeds—Pigeon pea, cowpea, beans	HHP (200–600 MPa, 20°C, 5 min, 10% slurry)	↓ Protein solubility, EA and WHC; OHC, ES, LCG unaffected. Effect strongly dependent on bean type	Sosa et al. (2020)
*Studies assessing food applications*			
Flour—Chickpea (roasted or not)	HHP (600 MPa, 50°C, 15–25 min)	Ready to eat gels produced. Treatment increased *G*′ and *G*″ but longer pressurization time decreased *G*′, *G*″, and gel firmness.	Alvarez et al. (2017)
Flour—Chickpea, pea, soy	HHP (350 MPa, 10 min, 20°C, 50% slurry)	Breadmaking with 42% wheat substitution. ↓ Moisture, loaf volume, and starch digestibility; ↑ crumb hardness	Collar and Angioloni (2017)

Abbreviations: EA, emulsion activity; ES, emulsion stability; FC, foam capacity; FS, foam stability; *G*′, storage modulus; *G*″, loss modulus; LGC, last gelation concentration; OHC, oil‐holding capacity; RS%, resistant starch; To, onset gelatinization temperature; Tp, peak gelatinization temperature; WAI, water adsorption index; WHC, water‐holding capacity; WSI, water solubility index; ∆*H*, gelatinization enthalpy.

Unless otherwise stated all comparison are done against untreated flour as control.

Nevertheless, the effects on functional properties when treating whole seeds were generally smaller compared to those found by other authors working on flours (Table 4). This is consistent with the findings of Alvarez et al. (2014, 2015), who observed smaller effects on gelling properties when comparing dry‐treated flours to slurries (bloom strength varied by only 5% in dry‐treated samples, while *G*′ of wet‐treated flour increased by up to 80%). Furthermore, higher flour concentration in such slurries again decreased the HHP‐induced effects (600 MPa decreased final pasting viscosity by 97% at 16.7% concentration, compared to a 51% decrease at 33% concentration). While the authors do not pinpoint an exact reason, they suggest this could be linked to the plasticizing effect of water (Alvarez et al. 2014). In other words, if more water molecules are available, improved interaction between water and molecules is possible, a phenomenon necessary to achieve disruption of starch crystalline regions, which underlies gelatinization. These results could be supported by the work of Kweon et al. (2017) on soy protein denaturation under pressure in solutions with different plasticizers. They observed that the presence of a high sugar concentration (50%) or other solvents helped reduce protein sensitivity to pressure.

Generally, researchers agree that HHP can induce partial starch gelatinization (∆*H* reduction in the 31%–67% range at 450 MPa among different pulses and studies), loss of crystallinity, and concomitant protein denaturation, especially at higher pressures such as 600 MPa (Kweon et al. 2017; Lin and Fernández‐Fraguas 2020; Z. Liu et al. 2021). These effects depend on the flour condition (amount of water present in the slurry) and also on interaction with other components, since the same treatment was found to be more effective on isolated starch compared to whole mung bean flour. Specifically, when assessing the crystallinity of starch upon HHP, it emerged that starch grains present in the flour retained more crystallinity compared to isolated starches, a similar trend observed with the preservation of Maltese cross and shape from microscopy observation. While the mechanism remains unclear, it appears that the presence of adhering protein bodies and the nonstarch matrix present in the flour has a protective effect against pressure (Z. Liu et al. 2021).

Another consistent finding across studies is an increase in batter viscosity and consistency upon pressurization treatments, while upon heating (e.g., during pasting analysis), the formed gels tend to be weaker, with lower viscosities or storage moduli (*G*′ after gelation reaching 227–253 Pa after 400 MPa compared to 566 Pa for untreated chickpea flour, Alvarez et al. (2016)). This behavior could result from altered protein conformation allowing better protein–protein interaction, along with partial starch gelatinization, both of which can increase viscosity (*G*′ increased from 13 mPa s up to 20–24 mPa s after HHP, Alvarez et al. 2016). Upon heating, the presence of predamaged starch can impede proper swelling, thus reducing the overall peak viscosity observed (Alvarez et al. 2014, 2016; Angioloni and Collar 2013; Lin and Fernández‐Fraguas 2020; Z. Liu et al. 2021).

Finally, there is agreement regarding the effects of the main processing variables: pressure, temperature, and holding time. Results suggest that target pressure is the main driver behind molecular conformation and functional property changes, while temperature and holding time play a minor role, although their effects are still visible, with gelatinization and protein denaturation increasing for longer holding times or higher processing temperatures (Alvarez et al. 2016; Kweon et al. 2017; Lin and Fernández‐Fraguas 2020; Z. Liu et al. 2021).

### 4.2 Dynamic High‐Pressure Processing

Dynamic high‐pressure treatments are widely used in the food industry, primarily for emulsification and stabilization of milk fats. These treatments involve a continuous process in which a liquid is forced through a narrow valve, creating high pressure, turbulence, and shearing forces that reduce the size distribution of emulsions and suspensions (Levy et al. 2021). The two main processes in this category are high‐pressure homogenization (HPH) and microfluidization, which differ mainly in the design of the treatment chamber. HPH features an impact ring after the narrow valve, while microfluidization splits the fluid into two capillaries, resulting in jet stream collision. The mechanisms believed to be responsible for macromolecule modification are high shear forces combined with cavitation and local temperature rise, which can lead to disaggregation of subunits (loss of quaternary structure) and partial loss of secondary and tertiary structures in proteins (Sahil et al. 2022). In starches, these effects are thought to arise from HPH treatment impacting both the molecular and granule structure. Several studies have highlighted the appearance of rough surfaces and holes on the granule surface after treatment, along with granule coalescence and loss of crystallinity. These phenomena, combined with molecular impacts such as amylopectin debranching and molecular weight reduction, have been associated with changes in macroscopic properties such as lower pasting curves, reduced retrogradation, and higher digestibility (S. Ma et al. 2024; Sahil et al. 2024; Zhu 2021). Interestingly, the improvement in functional properties observed in starches appears to be proportional to the pressure and number of passes (Zhu 2021), whereas this trend is not observed for proteins. In the latter case, some studies indicate optimal treatment conditions, beyond which functional improvements (e.g., solubility) may decrease, possibly due to excessive denaturation and subsequent aggregation of the protein molecules (Sahil et al. 2022).

Regarding application on flours, only a few studies are available (results are reported in Table 5), showing with excellent agreement a reduction in particle size upon treatment, with effects visible on cotyledon cells and on the amount of protein bodies attached to the starch granule (Adjei‐Fremah et al. 2019). The same authors observed a clear effect on OHC (50%–60% increase in all cultivars), while effects on WHC were much more subtle and dependent on the cultivar (ranging from 1% to 15%). Overall, the modifications in functional properties were suggested to result from both protein conformational changes (increased protein solubility) and structural changes in the degree of rupture of cell walls and seed coat. Interestingly, while no study so far has directly compared the effect of the same treatment on different pulses, the work of Adjei‐Fremah et al. (2019) assessed different cultivars of cowpea. Results vary according to the property assessed, with one cultivar being consistently affected (in WHC, OHC, and swelling), while another showed significant improvements only in OHC.

**TABLE 5:** Studies assessing dynamic high‐pressure treatment (HDPT) on pulse flours.

Form—Pulse type	Treatment type	Key results	References
Flour—Soy	HPH (100–150 MPa, two to three cycles, 13% slurry, 4°C)	↓ Protein solubility (but higher compared to heat treated); ↑ SFSH	H.‐H. Liu and Kuo (2016)
Flour—Soy	HPH (20–80 MPa, one cycle, 20% slurry)	↓ Particle size; ↑ adhesiveness at 20 MPa	Y. Zhang et al. (2021)
Flour—Three cowpea cultivars	Microfluidization (137 MPa, two cycles, Z chamber, 10% slurry, 25°C)	↓ Particle size; ↑ OHC. WHC and swelling affected differently form cultivars	Adjei‐Fremah et al. (2019)
Flour—Chickpea	HPH (40 MPa, 24% slurry, one cycle)	↓ Particle size; ↑ initial viscosity. In combination to heating improved rheological properties	Huang et al. (2022)
*Studies assessing food applications*			
Flour—Soy	HPH (100–150 MPa, two to three cycles, 15%–20% slurry)	Used for tofu formulation. ↓ Syneresis and particle size; ↑ hardness. Comparable quality attributes to heat‐treated soy flour	H.‐H. Liu et al. (2013)

Abbreviations: OHC, oil‐holding capacity; SFSH, surface free sulfuryls group; WHC, water‐holding capacity.

Unless otherwise stated all comparison are done against untreated flour as control.

While the authors provide an array of possible explanations, it is interesting to observe that the cultivar with the least improvement in functional properties was the one with a colored seed coat, thus richer in polyphenols. Since SDS‐PAGE results showed that only that specific cultivar presented a marked loss of soluble proteins (in the 50 kDa range), it could be inferred that the polyphenols released by the microfluidization treatment bound with proteins, creating insoluble complexes that eventually negatively impacted also the functional properties.

Regarding viscosity and gelling ability, Huang et al. (2023) observed that 40 MPa treatment induced minor viscosity improvement in treated chickpea flour slurry (consistency factor *K* from flow curve increased from 7 to 17 mPa s after HPH). However, when the product was heat treated after homogenization, the resulting gel showed increased viscosity (consistency factor rising from 31.6 to 53.3 Pa s) and *G*′ doubling in the frequency sweep test (from 53 to 103 Pa at 50 Hz, 8% slurry concentration), suggesting a synergistic effect, which the authors possibly ascribe to increased water competition from newly denatured proteins affecting starch hydration.

As a more direct food application, HPH has been used on soy flour as a pretreatment for tofu production (H.‐H. Liu et al. 2013; H.‐H. Liu and Kuo 2016). The authors observed that the resulting product had comparable quality to tofu made with thermally treated flour, with overall lower hardness (ranging from 86 to 204 g compared to 238 g for control) and decreased syneresis (from 3.1% to 7.9% compared to 9.98% in control). Interestingly, increasing the number of passes and the pressure applied (two to three cycles, 100–150 MPa) increased hardness, making the tofu more similar to the heat‐treated one. This modification was explained by a combination of disaggregation of insoluble protein bodies and exposure of more lipophilic regions, which allowed for stronger protein–protein interactions and thus better gel formation.

### 4.3 Ultrasound

US technology involves delivering sound waves through a medium (e.g., water) to the desired sample. This technology can be classified by the type of US used (high‐frequency, low‐intensity or low‐frequency, high‐intensity US) and by the setup (batch or probe systems; Bhargava et al. 2021). In both setups, the main mechanism behind the observed effects is thought to be cavitation. During sonication, waves traveling through the medium create alternating areas of high and low pressure. In low‐pressure zones, tiny vapor‐filled bubbles can form, grow, and eventually collapse. This collapse can lead to localized, temporary increases in temperature and pressure (2000–5000 K and 1800–3000 atm), along with strong turbulence and liquid jets responsible for the effects observed in the treated product, known as the “hot‐spot theory” (Mason and Peters 2002).

Although few studies have focused on the application of US to pulse flour so far (results reported in Table 6), much more research has been conducted on isolated starches and proteins. This growing interest is closely related to the increasing importance of pulses as sustainable protein sources in the global food system. In 2020, global pulse production reached approximately 91 million tonnes, with beans, chickpeas, and peas representing the major cultivated species (FAO 2025). Since pulse flours are particularly rich in starch and proteins, understanding the effects of US treatment on these macromolecules is essential for improving their technological functionality and expanding their use in food formulations. Hydrogen and disulfide bonds in proteins can be disrupted by US, leading to changes in secondary and tertiary structure, and resulting in denaturation, disaggregation, or aggregation of protein molecules. These phenomena underlie the observed improvements in solubility, water and oil binding, and rheological properties (Gharibzahedi and Smith 2020; Wang et al. 2020). In starches, the grain structure can develop cracks and pores on the surface after treatment, and crystallinity can also be affected. On the molecular level, more discordant data are presented, but starch debranching and molecular weight reduction have been reported. Overall, these effects on starch structure can induce changes in functional properties such as pasting, retrogradation, swelling, and solubilization (Awais et al. 2025; Zhu 2015).

**TABLE 6:** Studies assessing ultrasound treatment on pulse flours.

Form—Pulse type	Treatment type	Key results	References
Seed—Mung bean	US (25 kHz, 41 W/L, 25°C)	↑ Pasting viscosity but unaffected pasting properties on isolated starch	Miano et al. (2016)
Seed—Chickpea	US and germination (40 kHz, 10 min, 27°C)	↑ WHC, OHC, and solubility; ↓ gel firmness	Choudhury et al. (2024)
Seed—Chickpea	US (45–108 W/cm^2^, 10–30 min, 20°C)	↑ Hydration, pasting temperature and pasting viscosity. Highest increment for lower energy and shorter times	Bender et al. (2024)
Flour—Cowpea	US (20 kHz, 500 W, 20%–100% amplitude, 7.5–60 min, 50% duty cycle, 35°C)	↑ Peak viscosity, ∆*H*, WHC, OHC, and solubility; ↓ final viscosity	Dietz et al. (2025)
Seed—Navy bean	US (47 kHz, 750 W, 16°C)	↑ Pasting viscosity; ↓ pasting temperature	Ghafoor et al. (2014)
Seed—Mung bean, lentil	US and subsequent germination (40 kHz, 30% amplitude, 4°C, 10–20 min)	↑ Pasting viscosity; ↓ *G*′ and *G*″ compared to unsonicated but germinated flour. WHC, OHC, FC either increased or decreased according to pulse variety	Banura and Singh (2023)
*Studies assessing food applications*			
Seed—Lupin	US (20 kHz, 200 W, 25 min/4 h after boiling, 25°C)	Breadmaking: ↑ Loaf volume; ↓ hardness	Yaver and Bilgiçli (2021b)
Seed—Lupin	US (20 kHz, 200 W, 25 min/4 h after boiling, 25°C)	Pasta making minor decrease in firmness and volume, overall quality unchanged comparing to pasta made with untreated lupin flour	Yaver and Bilgiçli (2021a)
Seed—Black lentil	US and germination (35 kHz, 10 min, 20°C)	No negative impact on cookies formulation but lower ANFs and higher nutrition	Levent and Aktas (2024)

Abbreviations: ANFs, antinutritional factors; FC, foam capacity; *G*′, storage modulus; *G*″, loss modulus; OHC, oil‐holding capacity; WHC, water‐holding capacity; ∆*H*, gelatinization enthalpy.

Unless otherwise stated all comparison are done against untreated flour as control.

Interestingly, most US applications on pulses have focused on improving seed hydration (Bender et al. 2024; Ghafoor et al. 2014; Miano et al. 2016) or in combination with germination (Banura and Singh 2023; Choudhury et al. 2024; Levent and Aktas 2024), with functional properties assessed only as a secondary step.

Dietz et al. (2025) optimized US treatment to improve swelling power and solubility in cowpea flour. Results showed that WHC increased from 0.60 to 0.75 mL/g and OHC from 1.02 to 1.75 mL/g after US treatment, although no starch grain damage was observed (neither by SEM nor enzymatic assay). In contrast, increased soluble fiber content was reported, along with a marked reduction in particle size, suggesting these changes might play an important role. A minor effect on starch is also reported by Miano et al. (2016), showing that pasting properties of extracted starches did not change, while, when assessing whole mung bean flour, US treatment nearly doubled final viscosity (from 498 to 902 mPa s), suggesting that the US effect may be linked to specific effect on other components (such as proteins or fibers) or a possible interaction of them. Improvements in pasting viscosity upon US treatment have also been observed in chickpea (Bender et al. 2024), navy bean (Ghafoor et al. 2014), and mung bean (Banura and Singh 2023), nevertheless, due to the different treatment parameters used a proper comparison regarding the susceptibility of different pulses to US seems unfeasible. Notably, Bender et al. (2024) observed that all US‐treated samples showed higher viscosity during pasting compared to the control; however, the highest improvements were achieved with shorter treatment times. Peak viscosity at 30 min was 5%, 17%, and 10% lower compared to 10 min at 46, 81, and 109 W/cm^−2^ power intensity, respectively, and with lower energy delivered. This suggests that excessive cavitation forces may have a detrimental effect on pasting properties. This detrimental effect may occur because, above a specific power threshold, starch granules can be affected, reducing pasting properties. Unfortunately, available studies do not always report all treatment conditions in terms of actual power or amplitude delivered, which is necessary to properly compare different treatments.

Upon germination, Choudhury et al. (2024) observed increases of 11% and 3.5% for WHC and OHC, respectively, in sonicated chickpea flour, with no significant differences observed when US application was performed before or after soaking. Such a result is interesting because it shows that effective US treatment can be performed with no prior soaking, thus shortening the processing time in industrial context. In their study, gels obtained from US‐treated flour showed slightly lower consistency (from 66.3 to 64.6 g/s) and firmness (from 9.1 to 8.5 N) compared to control, possibly due to higher enzymatic activity during germination induced by greater cell disruption from cavitation. Similar results were also observed by Banura and Singh (2023) on lentils and mung bean, where *G*′ and *G*″ of flour–water dough decreased upon US treatment (*G*′ after 15 min US showed an average decrease of 38.7% and 27.5% for mung bean and lentil varieties, respectively, compared to germinated control).

Few studies assess the use of US‐treated pulse flour in food formulations (Levent and Aktas 2024; Yaver and Bilgiçli 2021a, 2021b). In pasta and cookie production, authors observed no significant differences in final product quality between US‐treated and untreated pulse flour. However, US either reduced the amount of ANFs present (−31.8% phytic acid compared to germinated flour Levent and Aktas 2024) or improved the nutritional profile, supporting the use of US‐treated products. In breadmaking, the use of US‐treated lupin flour not only improved nutritional properties but also produced breads with lower hardness (11.7 vs. 9.7 N) and higher loaf volume (2.72 vs. 3.12 mL/g) compared to untreated lupin flour, indicating the potential application in the sector. Nevertheless, these properties remained far from those of nonsubstituted samples, which had 5.11 N hardness and 4.27 mL/g loaf volume (Yaver and Bilgiçli 2021b).

### 4.4 Cold Plasma

CP involves generating an electrified cloud composed of ions, electrons, radicals, UV photons, and excited molecules using an electric generator that delivers high voltage and high‐frequency current to two electrodes separated by a dielectric material. Plasma can be generated in various configurations, such as dielectric barrier discharges (DBDs), plasma jets, and corona discharges. The technology can also be classified by the working gas (e.g., air, nitrogen, argon) and the applied pressure (atmospheric or partial vacuum; Chizoba Ekezie et al. 2017; Thirumdas et al. 2015). While seeds and flours can be directly treated, recent studies increasingly focus on treating water with CP and then using it to further treat food products. In plasma‐activated water (PAW), plasma‐generated reactive species react to form secondary species, including hydrogen peroxide, peroxy nitrite, nitric oxide, nitrates, and nitrite ions. This water is characterized by low pH and variable stability and can be used to treat products by immersion (Thirumdas et al. 2018).

Regarding the applications of CP and PAW specifically to pulse seeds to enhance functional properties, there is limited literature available so far (results are reported in Table 7), with some studies assessing germination improvement (Asefi and Singh 2025), and thus functional properties of the flour are assessed only secondarily. In contrast, much more research has been done on specific isolated components such as proteins and starches, where the effects and underlying mechanisms are better understood (S. Ma and Jiang 2024; Sharath Kumar et al. 2024). Specifically, CP can induce either crosslinking or depolymerization in amylose and amylopectin chains, while etching on the grain surface can lead to rougher surfaces. On seed proteins, the reactive species formed during treatment can induce oxidation (reducing sulfhydryl groups) and crosslinking, while modifications involving amino acids residual groups can lead to significant conformational changes (tertiary and secondary structure), resulting in changes in functionality.

**TABLE 7:** Studies assessing cold plasma and PEF treatments on pulse flours.

Form—Pulse type	Treatment type	Key results	References
Seed—Lentil	PAW and germination (20 kV, 20 Hz, 1–15 min, air)	↑ Protein solubility and amylase activity	Asefi et al. (2025)
Flour—Pea	CP (DBD, 8.8 kV, 3 kHz, 10 min, 12 mm gap)	↓ Protein solubility; ↑ WHC and OHC	Bußler et al. (2015)
Flour—Horse gram	CP (10–30 kV, 5–25 min, 45% RH, 1.5 mm gap)	↑ WHC, OHC, FC, EC, and swelling	Patra et al. (2024)
Seed—Mung, azuki, and kidney bean	CP (5 W, 0–20 min, 25°C, argon, 5 mm gap)	↓ LOX activity and WSI; ↑ RDS and WAI	Y. Wu, Feng, et al. (2023)
Flour—Guar	CP (10–20 kV, 5–20 min, 70 mm gap)	↑ WHC, OHC, FC, EC, and pasting viscosity	Kheto et al. (2023)
*Studies assessing food applications*			
Seed—Faba bean	PEF and germination (0.3–0.7 kV/cm, 0.5–3 kJ/kg, 20 Hz, 20 µs, 1000 bipolar rectangular pulses)	Breadmaking, PEF treated and germinated flour had the smallest impact on bread quality reduction compared to untreated or single treatments.	Johnston et al. (2024)
Flour—Chickpea	PEF (1–2 kV/cm, 400–500 kJ/kg, 200 Hz, 30 µs, bipolar rectangular pulses)	↓ Δ*H* and syneresis; ↑ hardness and consistency of obtained gels	Drudi et al. (2025)

Abbreviations: EC, emulsification capacity; FC, foam capacity; LOX, lipoxygenase activity; OHC, oil‐holding capacity; RDS, ready digestible starch; WAI, water adsorption index; WHC, water‐holding capacity; WSI, water solubility index.

Unless otherwise stated all comparison are done against untreated flour as control.

Highlighting the different responses of various flour fractions to CP, Bußler et al. (2015) reported an increase in WHC and OHC upon CP treatment only in specific pea flour fractions, with the protein‐rich fraction being affected (13% and 16% increase after 10 min treatment for WHC and OHC, respectively), while the starch‐rich fraction did not show significant differences. This suggests higher protein sensitivity to CP modification. OHC increases have also been found in horse gram (up to 53% after 20 min, 20 kV, Kheto et al. 2023) and guar flour (up to 16% after 5 min, 20 kV, Patra et al. 2024), with longer exposure times and higher voltages inducing more pronounced effects. These results further confirm the effect of CP on proteins, since OHC is generally thought to be related to the amphipathic nature of proteins rather than interaction with starches.

The same two authors also assessed starch granule morphology by SEM, observing clear signs of starch grain damage with increasing time and voltage, consistent with reports for treatments on isolated starches (S. Ma and Jiang 2024), impacts that they suggest are the basis of the improved water‐holding properties and swelling power. Moreover, they observed signs of detachment between starch and protein bodies, suggesting strong starch surface effects of CP. This effect might be an important characteristic of CP treatment, as the detachment of protein bodies from starch granules is important, especially in pulse flour, where protein bodies can hinder starch swelling and gelatinization if not properly detached.

Confirming the effect of CP on starch in flours, Y. Wu, Feng, et al. (2023) also assessed CP treatments on various bean flours, highlighting higher WAI (increasing from 4.4% up to 20.8% depending on pulse type) and lower WSI (generally around 35%–38% reduction) upon treatment. These results matched the increase in RDS and a reduction in RS, suggesting either partial gelatinization or sufficient starch etching and damage to facilitate starch enzymatic digestion. This work is also the only one available comparing the effect of the same CP treatment on different pulses, allowing observation of the effect on different matrices. While the effects induced by CP were similar in trend, the intensity varied by botanical origin. Kidney bean WAI and starch digestibility were improved significantly more compared to mung and azuki beans. Although not proven, this different behavior was attributed to differences in composition (specifically total fibers and fatty acid composition) and different accessibility of the starch granule to the plasma reactive species.

### 4.5 Pulsed Electric Field

PEF technology involves the delivery of short (µs–ms), high‐voltage pulses of current through a conductive medium to create an electric field. High electric field strength is used for microbial inactivation, while low and medium electric field strengths are used in the food industry for applications involving mass transfer. When electric fields exceed a critical value, they can induce cell membrane permeabilization, enhancing the mass transfer process (Donsì et al. 2010; Karki et al. 2022).

More recently, PEF has been investigated for use on products where intact cell membranes are absent or damaged (e.g., flours or PIs) as a tool to improve technological properties, focusing on possible effects on the structural modification of macromolecules such as starch and proteins (Giteru et al. 2018). The underlying mechanisms in this application, though not fully understood, are believed to involve structural stretching and reorganization due to the polarization force of the electric field on charged structures, as well as electrochemical reactions such as charged particle and radical formation from water hydrolysis near electrode surfaces (Giteru et al. 2018; Milanezzi and Silva 2025). However, increasing specific energy inevitably produces more heat due to the Joule effect, so temperature‐mediated changes may also occur.

Table 7 summarizes literature results on the effect of PEF treatment on the functional properties of pulse flours. As shown, only two studies are available, highlighting the still limited knowledge in this area. Moreover, both studies focus on product application rather than directly assessing flour properties or the fundamental effect of PEF on them. In the study of Johnston et al. (2024) low specific energies and low electric fields (0.5–3 kJ/kg, 0.3–0.7 kV/cm) were used to avoid damaging the seed embryo, which is necessary for germination. Although flour obtained from treated seeds was later used in breadmaking trials, no functional properties of the flour itself were systematically evaluated. The authors only reported slight, nonsignificant improvements in bread texture parameters, including reduced hardness (from 14.2 to 12.7 N) and increasing cohesiveness (from 0.63 to 0.69) without proposing a mechanistic explanation. Therefore, the study provides only indirect indications regarding possible flour functionality changes following PEF treatment.

The second study (Drudi et al. 2025) explored the use of PEF‐assisted processing to produce chickpea flour gels with tailored consistencies. However, the high specific energy applied (400–500 kJ/kg) caused substantial Joule heating, which the authors identified as the primary driver of the observed structural modifications. The resulting increase in starch gelatinization and protein aggregation was therefore mainly associated with thermal effects rather than with purely nonthermal PEF mechanisms. Although slight differences in in vitro protein digestibility between PEF‐treated and conventionally heated samples suggested a possible secondary contribution of the electric field, no clear mechanistic effect of PEF on pulse flour components was demonstrated.

Since neither study assessed the application of higher electric fields, which are generally thought to be needed to achieve molecular modifications, it is currently impossible to describe the effects of PEF treatments on the functional properties of pulse flour components. More studies are needed to properly understand the effects of intermediate specific energies and higher electric fields on pulse flours and their possible applications. Nevertheless, possible effectiveness of the technology might be inferred from studies performed on cereal flours or other pulse components, as some studies have found that lower electric fields (< 5 kV/cm) can result in structural modification of flours, such as cassava (Conde et al. 2022), wheat (Achayuthakan et al. 2023), and oat (Duque et al. 2020), or on PIs (Gulzar et al. 2024) and concentrates (Melchior et al. 2020).

## 5 Conclusions

Pulse flours offer significant nutritional benefits, including high protein and fiber content, making them well‐suited for plant‐based and gluten‐free products. However, their broader use is limited by challenges such as undesirable beany off‐flavors, inconsistent hydration properties, antinutritional factors, and the absence of essential structuring agents like gluten. Addressing these limitations requires targeted functionalization strategies, which should be planned with consideration for the entire flour production process, acknowledging the impact of proper milling and any necessary stabilization steps (e.g., drying) if water is added during modification.

**FIGURE 4:**
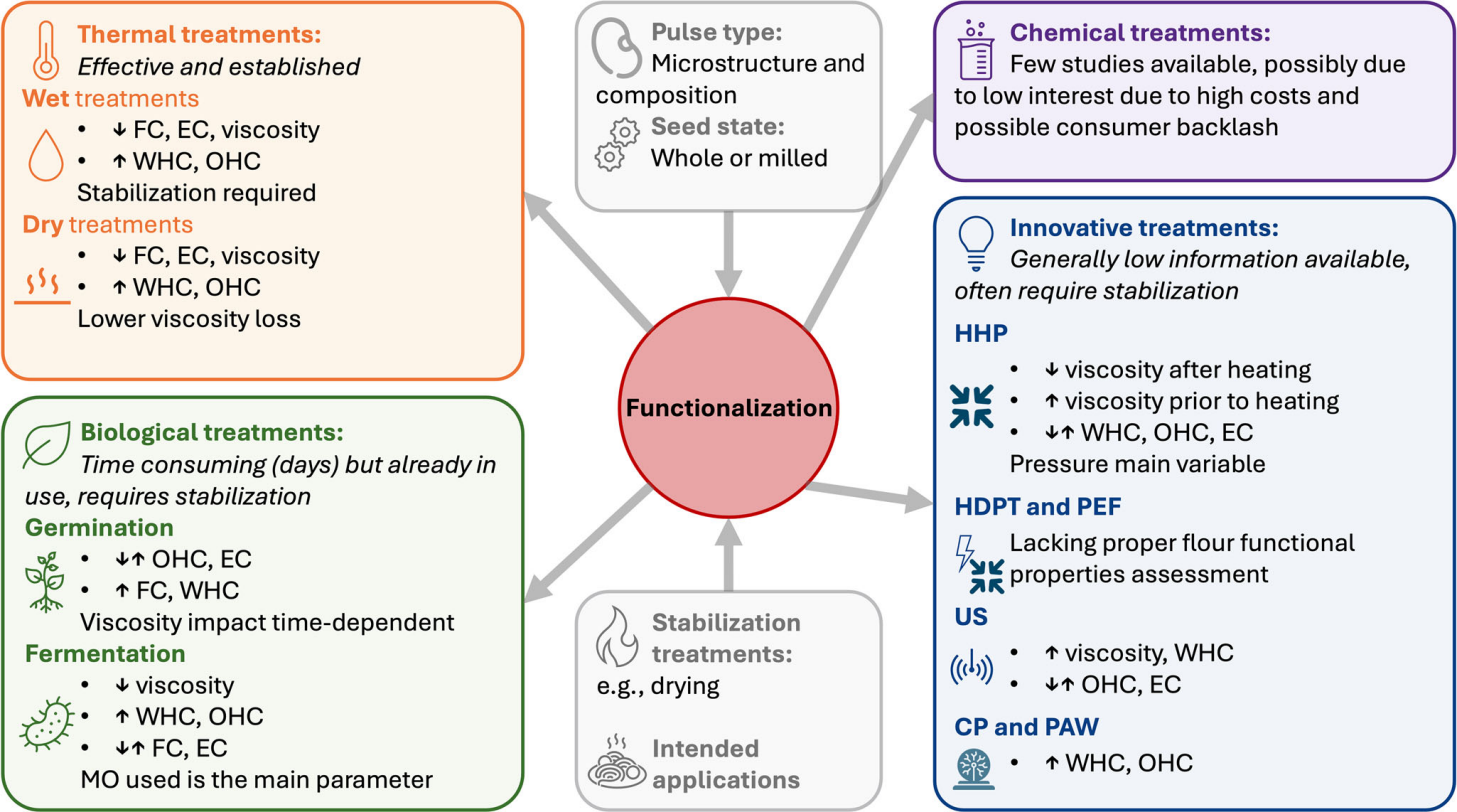
Schematic summary of the main effects of the discussed modification strategies on pulse flours. The symbols ↑, ↓, and ↑↓ refer to increases, decreases, or contradictory results, respectively. CP, cold plasma; EC, emulsion capacity; FC, foam capacity; HDPT, high dynamic pressure treatments; HHP, high hydrostatic pressures; MOs, microorganisms; OHC, oil‐holding capacity; PAW, plasma‐activated water; PEF, pulsed electric fields; WHC, water‐holding capacity.

Various modification techniques are available (summarized in Figure 4), each with specific mechanisms and resulting changes. Thermal methods, applied wet or dry, are the most studied and widely used. These methods consistently improve WHC and OHC across pulse types, though they often reduce foaming and emulsifying properties. Wet treatments facilitate faster heat transfer, significantly decrease pasting viscosities, and increase particle size, while dry treatments generally avoid complete starch gelatinization and have a milder impact on pasting viscosities. Both approaches have the advantage of not requiring new or expensive industrial equipment; however, wet treatments require a drying step to stabilize the flour, while roasting does not. Both options strongly reduce antinutritional factors, and roasting specifically has been reported to improve the aromatic profile and color.

Biological methods, including germination and fermentation, offer another approach by causing active compositional changes within the flour. Both treatments require a drying step to stabilize the product and, unlike thermal treatments, require longer processing times (often days), which must be considered for industrial applicability. Germination uses endogenous enzymes to weaken cotyledon structures, enhance hydration, and reduce ANFs. Studies consistently show that shorter germination periods can increase pasting viscosity, while longer periods decrease it. WHC and FC are consistently improved, while OHC and emulsion capacity show contradictory results. This variability in trends and degree of impact may result from differences in composition and microstructure of the pulse seeds, with more compact seeds such as faba beans being greatly affected. Fermentation significantly alters color, aroma, and chemical composition due to microbial metabolism. The choice of conditions (wet or solid state) and MO type is critical and impacts the results more than the pulse type used, so it should be aligned with the intended use of the modified flour. Studies consistently report improved WHC and OHC but reduced pasting viscosity due to starch degradation. Critically, most research includes sterilization steps on the pulses prior to fermentation, a step that significantly affects functional properties but is often overlooked in literature.

Chemical methods involving specific molecular modifications are mainly applied to isolated starch and protein fractions, primarily due to economic viability and consumer labeling concerns associated with whole flour treatments. These concerns are also reflected in the limited number of available studies, which is insufficient to draw solid conclusions about their effectiveness.

Innovative nonthermal techniques are increasingly being explored, though further research is needed to fully understand their mechanisms of action when applied to pulse flour. Specifically, there is a lack of studies on PEF, CP, and dynamic high‐pressure treatments, which currently only allow observation of promising preliminary applications. For instance, plasma treatment can impact functional properties with short treatments and has the advantage of not requiring subsequent drying. Moreover, some of the studies assessed used soy, which, although also a legume, has a markedly different chemical composition, making it difficult to directly translate such results to pulses. Regarding US applications, although studies are available, there is often a lack of proper reporting of treatment parameters, making comparison of results complex.

A key exception among innovative technologies is the use of HHPs, where multiple studies have shown that pressurization leads to partial starch gelatinization and protein denaturation, increasing batter viscosity but potentially resulting in weaker gels after heating. Consistent findings also show lower protein solubility after treatment, possibly explaining the frequently reported lower OHC. Different studies agree that the main processing variable is the pressure reached, with treatment time having only a slight effect on the product, thus allowing for faster application that will likely benefit industrial use. Nevertheless, better results are obtained when the seeds are milled prior to treatment and mixed with water, which also requires specific drying and stabilization steps.

To summaries, selecting the appropriate modification technique is critically important and should be based not only on understanding the underlying mechanism but also on the intended application of the modified product. This ensures the development of a better performing ingredient for the specific task and allows for assessment of the economic feasibility of the application.

## Author Contributions

**Federico Drudi**: conceptualization, writing – original draft, writing – review and editing, methodology, data curation, validation, visualization. **Silvia Tappi**: conceptualization, writing – review and editing, validation, supervision, methodology. **Urszula Tylewicz**: methodology, validation, writing – review and editing, conceptualization, supervision, funding acquisition.

## Conflicts of Interest

The authors declare no conflicts of interest.
